# *In vivo* model for microbial invasion of tooth root dentinal tubules

**DOI:** 10.1590/1678-775720150448

**Published:** 2016

**Authors:** Jane L BRITTAN, Susan V SPRAGUE, Emma L MACDONALD, Robert M LOVE, Howard F JENKINSON, Nicola X WEST

**Affiliations:** 1 - University of Bristol, Department of Oral and Dental Sciences, Bristol, United Kingdom.; 2 - University of Otago, Department of Oral Diagnostic and Surgical Sciences, Dunedin, New Zealand.

**Keywords:** Dentine, Root caries, Microbiology, Biofilms

## Abstract

**Objective:**

Bacterial penetration of dentinal tubules via exposed dentine can lead to root caries and promote infections of the pulp and root canal system. The aim of this work was to develop a new experimental model for studying bacterial invasion of dentinal tubules within the human oral cavity.

**Material and Methods:**

Sections of human root dentine were mounted into lower oral appliances that were worn by four human subjects for 15 d. Roots were then fixed, sectioned, stained and examined microscopically for evidence of bacterial invasion. Levels of invasion were expressed as Tubule Invasion Factor (TIF). DNA was extracted from root samples, subjected to polymerase chain reaction amplification of 16S rRNA genes, and invading bacteria were identified by comparison of sequences with GenBank database.

**Results:**

All root dentine samples with patent tubules showed evidence of bacterial cell invasion (TIF value range from 5.7 to 9.0) to depths of 200 mm or more. A spectrum of Gram-positive and Gram-negative cell morphotypes were visualized, and molecular typing identified species of *Granulicatella*, *Streptococcus*, *Klebsiella*, *Enterobacter*, *Acinetobacter*, and *Pseudomonas* as dentinal tubule residents.

**Conclusion:**

A novel *in vivo* model is described, which provides for human root dentine to be efficiently infected by oral microorganisms. A range of bacteria were able to initially invade dentinal tubules within exposed dentine. The model will be useful for testing the effectiveness of antiseptics, irrigants, and potential tubule occluding agents in preventing bacterial invasion of dentine.

## INTRODUCTION

Root caries, pulpitis, and dentine hypersensitivity are becoming increasingly more problematic as the dentate human population ages^17^. Gingival recession can lead to exposure of dentine and tooth wear can result in opening of dentinal tubules on the exposed surface. When dentine becomes exposed as a result of gingival recession, or through dental caries, cracks, or microleakage around restorations, microorganisms are able to gain access to the tubules^15^. More microorganisms are found in the dentine adjacent to periodontal pockets than in healthy radicular dentine, and more bacteria are found in superficial root dentine than in middle dentine^1^. Bacteria can also laterally invade the root surface along the incremental lines of cementum and then infiltrate the dentine^21^. Bacteria can penetrate through hypomineralized enamel into the dentine and contribute to pulpal pain symptoms of teeth with molar incisor hypomineralization^10^.

Bacterial persistence within the dentinal tubules may exacerbate development of root caries, and the formation of complex microbial communities within deeper dentine or root canal space^26^ may play a significant part in endodontic treatment failure^18^. Evidence suggests that the bacteria that initially invade dentinal tubules are often from the genera *Enterococcus* and *Streptococcus* ^15^. *Enterococci* in particular readily penetrate dentinal tubules^13^ ^,^ ^18^. Root dentinal tubule invasion models in vitro have been utilized widely to study different bacterial penetration capabilities^14^ and model pulpal infections^15^, and to test the effects of antimicrobials^3^ ^,^ ^4^ and irrigants^12^. The models have provided valuable information on the mechanisms involved in growth and penetration of dentine^5^, and the potential for various agents, such as photodynamic therapy^29^ to help with controlling infection. However, the various laboratory models usually incorporate dentine samples that are exposed to an artificial nutrient environment in order to achieve infection with relevant microorganisms. Under natural conditions, dentine would be exposed to salivary components, gingival fluid, immune system molecules, and potentially hundreds of different microorganisms^19^. Most *in vitro* dentine infection models employ conditions that are quite different from the natural *in vivo* infection environment.

Dentine studies *in vitro* have also included testing various compounds for ability to occlude tubules as desensitizing agents^2^. Valuable information has been obtained about the properties and effectiveness of such agents^27^, but these are only just beginning to be tested under suitable *in vivo* conditions. For example, West, et al.^28^ (2011) determined the abilities of desensitizing toothpaste technologies to occlude patent dentinal tubules in a clinical environment. Healthy subjects wore lower intraoral appliances retaining dentine samples, and these were analyzed after 4 d of treatment for degree of occlusion^28^. We have utilized the basis of that study to develop a model for microorganism invasion of dentinal tubules *in vivo*. This will provide a suitable platform by which to investigate bacterial invasion of dentine within a clinical environment, and to test for effectiveness of tubule-occluding or antimicrobial agents to prevent bacterial invasion of dentine.

## MATERIAL AND METHODS

### Root dentine

Non-carious, unrestored human canine or pre-molar teeth with single root canals were obtained from orthodontic extractions. Teeth were obtained with informed consent and the study was approved by Central and South Bristol Ethics Committee (REC ref. 04/Q2006/50). Following extraction, teeth were soaked in 2% sodium hypochlorite (NaOCl) for 48 h and any soft tissue remaining was removed. Prior to sectioning, roots were washed in copious amounts of water, and rinsing was repeated following sectioning to ensure no traces of NaOCl remained. Teeth were stored in sterile distilled H_2_O at 4°C until required. Roots were sectioned using a water cooled steel bladed cutting machine (Isomet Saw, Buhler Ltd., Evanston, IL, USA). In brief, the crown and root tip were removed, the remaining root was cut into 0.5 cm lengths, and the cervical segments were longitudinally sectioned in such a way that the root canal was exposed. The root sections were then autoclaved (121°C, 20 min) in distilled H_2_O, which did not visually affect tubule structures^5^, and stored at 4°C.

### Preparation of intra-oral appliances

**Figure 1:**
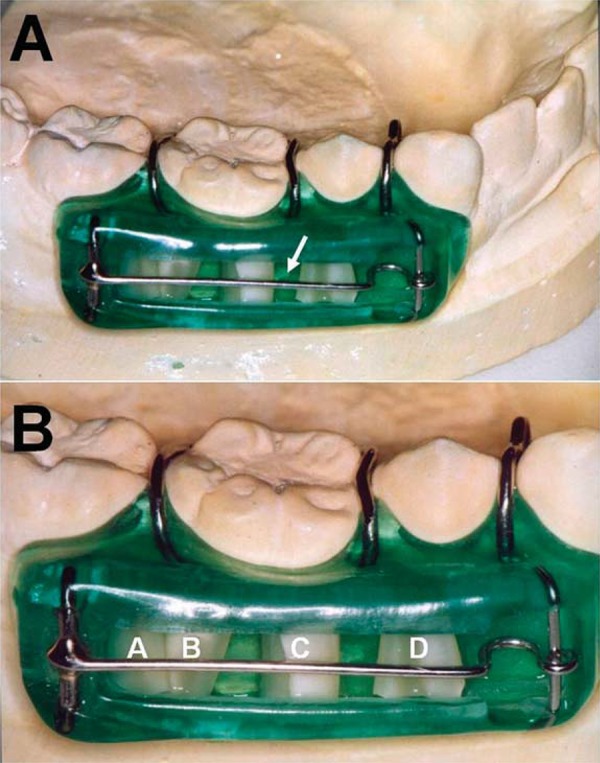
View of dental appliance in place on a dental mould (A) and a close-up view showing positioning of dentine samples (B). This shows the lower right appliance and the dentine pieces with pulpal faces outwards labelled A-D. A similar appliance was placed on the lower left, with four dentine pieces designated E-H. The dentine samples were retained with wax on the base side and with a metal retaining wire on the exposed side (arrowed in panel A). Appliances were custom made for each individual human subject

For each subject, a lower alginate impression was recorded in a perforated stock tray. Within 30 min the impressions were poured in Kaffir D dental stone and subsequently two lower-oral appliances were constructed from Forestacryl^®^ self curing acrylic (Pearson Dental Supply Co., Sylmar, CA, USA). Adams cribs were constructed to fit the mandibular first molars to aid retention and wire loops were constructed in an anterior and posterior trench region to hold the dentine samples in place (Figure 1). The cervical region root sections were mounted into the appliances in such a way that the buccal facing surface was flush with, or just below, the level of the surrounding acrylic surface. Before placement in the appliance, the root sections were dipped in sterile distilled water, the face to be in contact with the appliance was dried and the sample mounted onto a small drop of molten sticky wax within the trench of the appliance. Once all four sections were in place they were then further secured in position with the wire loop that was built into the appliance (Figure 1). The appliances were stored overnight at 4°C in a sterile airtight container containing damp tissue to prevent them drying out.

### Experimental design

Ethical approval for this work was obtained from the Institutional Ethical Review Board (REC ref. 04/Q2006/50). Inclusion criteria were healthy volunteers aged 18 or over that could accommodate a lower buccal appliance. Exclusion criteria were: pregnancy, lactation, gross caries, unstable periodontal disease, antimicrobial medication within 7 d previously, orthodontic appliances that would interfere with the study evaluations, and tongue or lip piercing. Four subjects, age range 20-26 years, with informed consent, were fitted with the appliances which were then worn over the course of the next 15 d between 09:00 and 21:00 h. They were removed for 1 h twice a day for mealtimes and also for the period over which any drink other than water was consumed. At 21:00 h, subjects removed the appliances, brushed them with tap water, rinsed them in running water for 20 s, and then stored them overnight in an airtight container. Subjects brushed their teeth morning and evening with fluoridated dentifrice. On completion of the trial on day 15, root samples were removed from the appliances at 15:00 h. There were no adverse events.

### Microscopic analysis of bacterial invasion

Six pieces of dentine from the appliances were fixed in 10% neutral buffered formalin for 7 d before being demineralized in 10% formic acid containing 2% formalin for 7 d. Samples were then dehydrated (70% IMS-denatured alcohol x2, 90% IMS x2, 100% IMS x3, xylene x3, paraffin wax x3) before being blocked in wax. Fifteen transverse sections, 6 mm thick and 60 mm apart from the next section, were cut from each dentine sample, mounted on poly-lysine slides, and heat fixed prior to staining using the Brown and Brenn method^6^.

Penetration of bacteria into dentine was visualized by light microscopy at x400 magnification. The central point of the root section was identified and five fields of view radiating out from this point were examined for each of the 15 root sections. The extent of invasion was initially expressed as the tubule invasion index (TI)^16^, where 1 to 20 tubules (*per* field) invaded scored 1; 21-50 tubules invaded scored 2; and >50 tubules invaded scored 3. These scores were then converted to Tubule Invasion Factor (TIF) that took depth of invasion of tubules into account^5^. The TIF was obtained by multiplying the TI by the invasion depth score: x1, where invasion depth was ≤50 mm; x2, where ≥5 tubules *per* field showed invasion depth >50 mm; and x3, where ≥5 tubules per field were invaded to depth of ≥100 mm, as previously described ^5^.

### Identification of bacteria

Two root pieces from each appliance were rinsed in sterile distilled water and stored at -80°C. One of the specimens in each case was used to optimize the methods for DNA extraction and amplification. Once this had been achieved, the second sample from each subject was transferred into a microfuge tube containing 0.1 mL sterile 10% EDTA (pH 6.5), vigorously vortex-mixed, and incubated at room temperature for 30 min to partially decalcify. Samples were then transferred into 0.1 mL 2 M citric acid (pH 1.6) and incubated for 30 min. Samples were extensively rinsed in sterile distilled H_2_O and transferred to tubes containing Gene Releaser (Cambio, Cambridge, Cambs, UK) for DNA extraction according to the manufacturer’s instructions.

**Figure 2:**
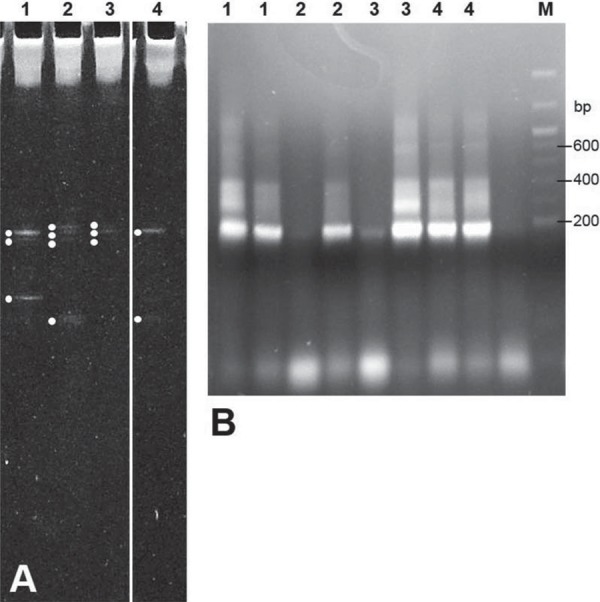
DGGE gel of DNA samples extracted from four (1-4) root dentine blocks (A) and agarose gel (B) showing PCR products derived from two selected DGGE gel bands from each sample (1-4). DNA bp markers (M) are indicated

The DNA extracts were used as templates in Polymerase Chain Reaction (PCR) amplification with universal 16S rRNA gene primers: DGGE F3(5’CGCCCGCCGCGCGCGGCGGGCGGGGCGGGGGCACGGGGGGCCTACGGGAGGCAGCAG) with the GC clamp for Denaturing Gradient Gel Electrophoresis (DGGE)^25^ and R2 (5’ATTACCGCGGCTGCTGG), to amplify a product of 160 bp. The presence of correct sized fragments was confirmed by agarose gel electrophoresis. Subsequently, aliquots (6 mL) of PCR products were subjected to DGGE (50-60% denaturant gradient) and the separated bands were ethidium bromide-stained and visualized under UV light (344 nm) (Figure 2A). Bands were excised from the gel lanes, transferred to tubes containing 0.3 mL TE buffer (5 mM Tris-HCl, 10 mM EDTA, pH 7.5) and the DNA was allowed to elute from the gel fragments for 16 h at 4°C.

Each of the eluted gel bands was then subjected to further PCR amplification using primers F3 (5’CCTACGGGAGGCAGCAG) (as above minus GC clamp), and R2 (above). Presence of amplified fragments was confirmed by agarose gel electrophoresis (Figure 2B). The fragments were gel-purified (QIAquick PCR Purification Kit, Qiagen, Manchester, Lancs, UK), ligated into plasmid pCR2.1 (Invitrogen, Thermo Fischer Scientific Inc., Waltham, MA, USA) and transformed into *Escherichia coli* XL1-Blue by standard procedures. Plasmids were extracted from transformant colonies using QIAprep Spin MiniPrep Kit (Qiagen), checked by agarose gel electrophoresis, and the 160-bp inserts were dideoxy-sequenced (Geneservice Ltd., Cambridge, Cambs, UK). The partial 16S rRNA gene sequences were then compared with 16S rRNA gene sequences in GenBank using the standard nucleotide NCBI/BLAST program.

## RESULTS

### Microscopic analysis of bacterial invasion

**Figure 3:**
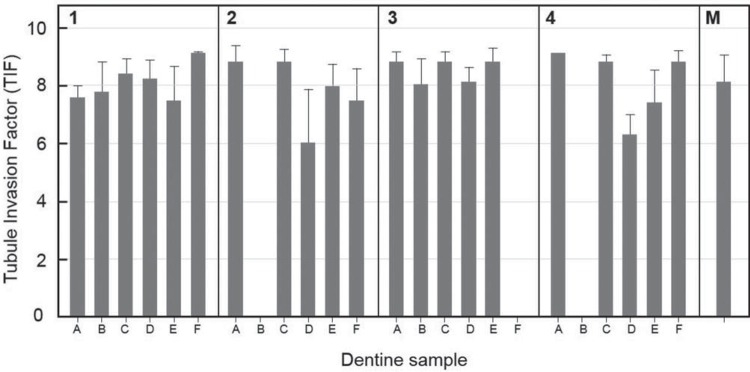
Mean level of microbial cell invasion into dentine samples carried by four subjects (1-4). Columns A through F correspond to dentine samples (see Figure 1 legend). Invasion is expressed as Tubule Invasion Factor (TIF) (see Material and Methods) which takes into account numbers of tubules containing bacteria and depth of penetration. Samples B, F, and B in subjects 2, 3, and 4 respectively, were not analyzable because of deformed dentine structures. Panel 5, combined dataset mean±standard deviation (8.09±0.87). Error bars are ± standard deviation from microscopic analysis of 75 individual sections (n=21)

The tooth root dentine pieces were mounted into appliances as shown in Figure 1. The samples were designated A-D (lower right) (Figure 1B) and E-H (lower left). After 15 d *in vivo*, the root pieces were removed and processed as described in Material and Methods for microscopic analyses (A-F). All samples containing patent dentinal tubules showed high levels of bacterial invasion, with TIF values in the range of 5 to 9 (Figure 3). Three dentine samples (Figure 3) could not be assessed for invasion because of disintegration of internal dentine structure.

The histochemical staining profiles with respect to type of organisms present within the dentinal tubules, pattern of tubule penetration, depth of invasion, and surface adhesion (biofilm) varied enormously across the dentine samples. We examined all dentine samples to confirm bacterial invasion, but in the following descriptions we have included only representative micrographs exhibiting distinct features of the invasion processes.

**Figure 4:**
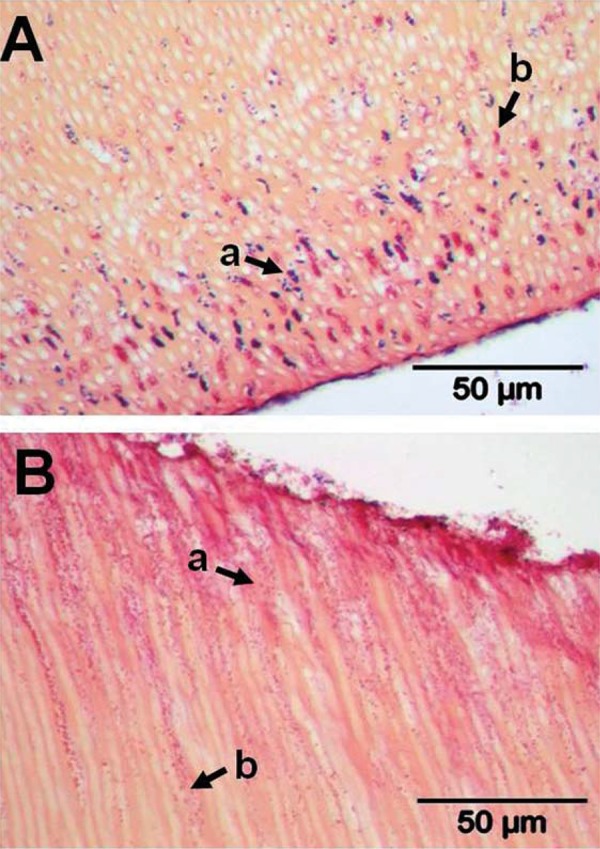
Transverse sections of human roots after 15 days incubation *in situ* in subject 1. Sections were prepared as described in Material and Methods, and stained by Brown & Brenn method. Panels: A, sample D, Gram-positive bacteria (a) and Gram-negative rods (b) penetrated to ~100 mm; B, sample F, small cocci infiltrated throughout the dentinal tubules appearing Gram-positive (a) towards the outside and Gram-negative (b) more internally. TIF scores for specimens are shown in Figure 3

For subject 1, root sample D, there was invasion of purple-stained Gram-positive cocci (Figure 4A, arrowed a) and pink-stained Gram-negative rods (Figure 4A, arrowed b) to depths of >100 mm. Root sample F, on the other hand, seemed to be entirely permeated by small cocci bacteria. These stained Gram-positive in areas of denser colonization (Figure 4B, arrowed a) or Gram-negative in regions of deeper (~150 mm) invasion (Figure 4B, arrowed b). This Gram-variable staining was seen previously in laboratory studies of dentine invasion by pure cultures of streptococci^16^.

**Figure 5:**
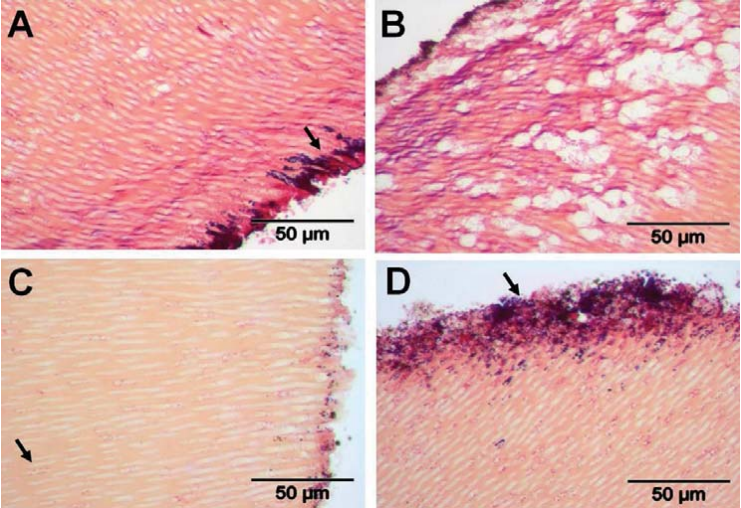
Transverse sections of human roots after 15 days incubation *in situ* in subject 2. Sections were prepared as described in Material and Methods, and stained by Brown & Brenn method. Panels: A, sample A, Gram-positive cocci invading to a depth of >150 mm and showing a 10 mm depth dense biofilm at the surface (arrowed); B, sample B, disintegration of internal dentine structure meant that sections from this sample could not be analyzed; C, sample C, Gram-positive bacteria at the surface and deep penetration by smaller Gram-negative bacteria ≥200 mm (arrowed); sample D, Gram-positive and Gram-negative bacteria penetration with accumulation of Gram-positive cocci biofilm at the surface of the sectioned sample (arrowed). TIF scores for specimens are shown in Figure 3

In subject 2, sample A carried a dense invasive biofilm of Gram-positive stained material at the surface (Figure 5A) and there was invasion of tubules >150 mm by Gram-positive cocci. A similar pattern was seen for root sample D (Figure 5D), while sample C from subject 2 showed Gram-positive bacteria at the surface and deep penetration ≥200 mm by small Gram-negative organisms. Block B from this subject was one of the samples that could not be properly analyzed, as the internal dentine structure was disintegrated (Figure 5B).

**Figure 6:**
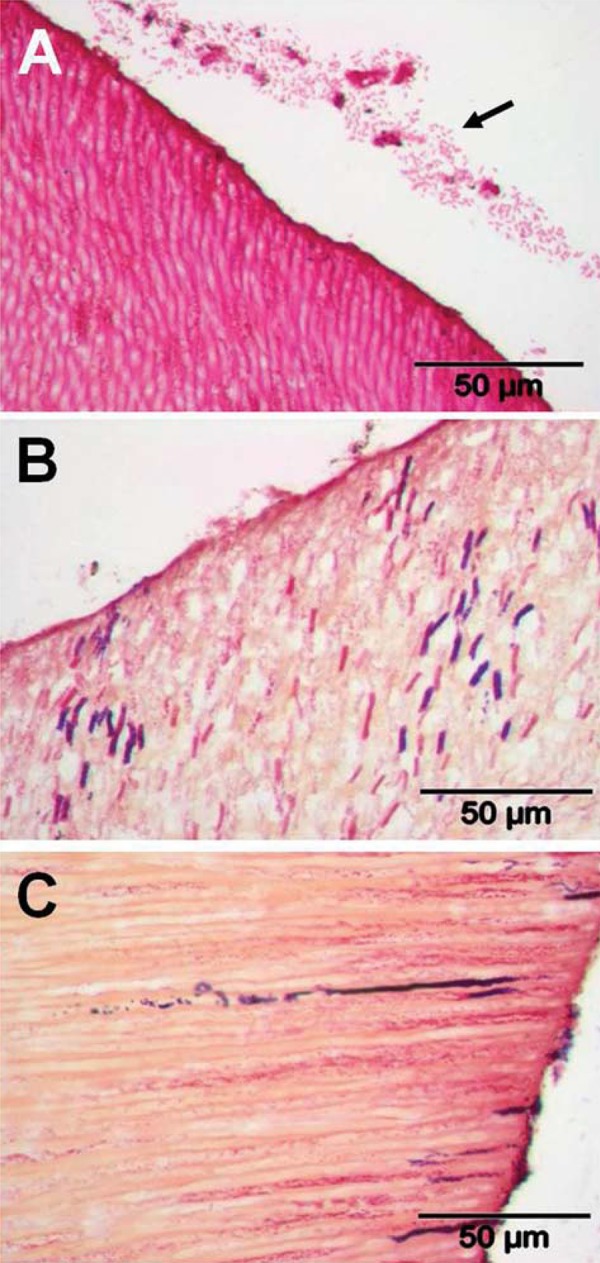
Transverse sections of human roots after 15 days incubation *in situ* in subject 3. Sections were prepared as described in Material and Methods, and stained by Brown & Brenn method. Panels: A, sample C, invasion by Gram-negative rod-shaped bacteria, with a strip of Gram-negative rods ~30 mm from the surface (arrowed); B, sample D, larger Gram-positive and Gram-negative rods (~5 mm length) well-separated but penetrating ≥150 mm; C, sample E, individual tubules appear to show long lines of invading Gram-positive and Gram-negative bacteria. TIF scores for specimens are shown in Figure 3

Figure 6 shows sections from blocks C, D, and E from subject 3. Sections through C (Figure 6A) contained small Gram-negative rods within the dentinal tubules. A band of Gram-negative rods was present a small distance away from the surface of the dentine sample, perhaps having been present on the dentine surface prior to sectioning (Figure 6A). Sample D contained Gram-positive and Gram-negative rods (~5 mm length) in well-separated tubules and penetrating ≥150 mm (Figure 6B). In sample E, individual tubules contained deep lines of invading Gram-positive and Gram-negative bacteria (Figure 6C).

**Figure 7:**
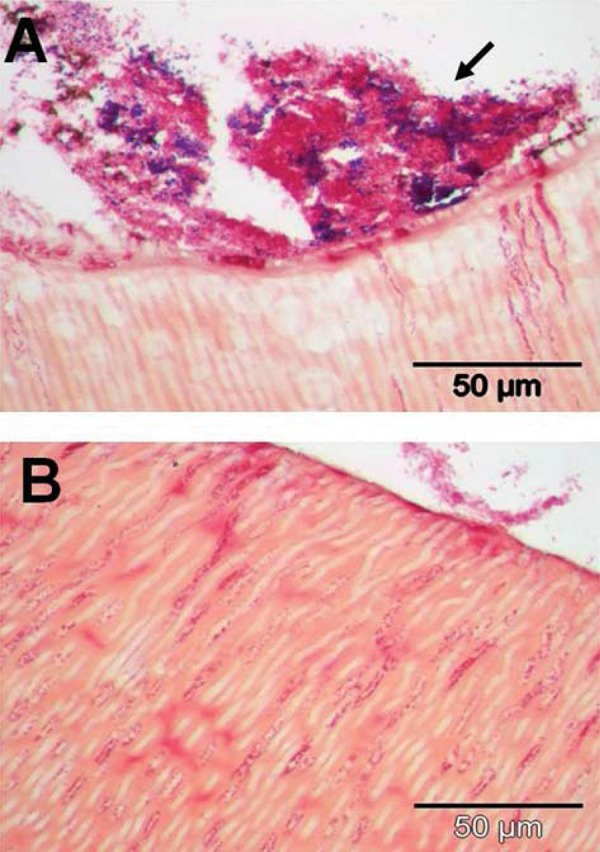
Transverse sections of human roots after 15 days incubation *in situ* in subject 4. Sections were prepared as described in Material and Methods, and stained by Brown & Brenn method. Panels: A, sample D, shows invasion by Gram-positive cocci and Gram-negative rods, together with a thick biofilm on the surface (arrowed) comprised of Gram-positive cocci and matrix material, staining pink; B, sample E, penetration by groups of Gram-positive cocci. TIF scores for specimens are shown in Figure 3

From subject 4, sample D showed invasion by Gram-positive cocci and Gram-negative rods, and a dense biofilm on the surface comprised of Gram-positive cocci and matrix material (Figure 7A). Sample E showed distinct penetration of tubules by groups of Gram-positive cocci (Figure 7B).

These results demonstrated that the dentine samples mounted onto the appliances were all readily susceptible to infection by invading microorganisms. The technique therefore was a very effective mean of achieving invasion of dentinal tubules by a variety of different oral bacteria.

### Molecular identification of invading bacteria

Within one of the dentine samples selected at random from subject 1 (3 DGGE bands, Table 1) we identified principally Gram-negative bacteria including *Klebsiella pneumoniae*, *Enterobacter* species, *Enterobacter hormaechei,* and sequences similar to those from some uncultivated bacteria from faeces (Table 1). There was 100% sequence match over 160 bp to *E. hormaechei*. Subject 2 sample contained only Gram-negative *Klebsiella*-like bacteria, while subject 3 (three DGGE gel bands) provided sequences 100% identical to *Acinetobacter* and *Streptococcus* database entries, and 99% to *Enterobacter* spp. (Table 1). The root sample from subject 4 provided sequences with 100% matches to Gram-positive bacteria *Granulictaella*, *Streptococcus mitis*, *Streptococcus oralis*, and *S. gordonii*, and to *Pseudomonas* species and uncultivated organisms. Overall, these analyses showed a diversity of bacterial infection to a degree similar to morphological varieties of Gram-negative and Gram-positive organisms visualized microscopically (Figures 4-7).

**Table 1:** Microorganisms identified from within dentinal tubules following DNA extraction, PCR, DGGE, and 16S rDNA sequencing. The partial 16S rRNA sequences were compared using BLASTN with 16S rRNA gene sequences within GenBank

Subject	DGGE band	GenBank description1	No. 100% matches^2,4^	GenBank entry example^3^
1	U1	*Klebsiella pneumoniae*	0	KP297466
		*Enterobacter hormaechei*		KP027682
	U2	*Enterobacter spp.*	499	KP091277
		*Enterobacter hormaechei*		KF516241
	L2	Uncultivated from faeces	>500	KF841982
2	U1	*Klebsiella oxytoca*	499	CP004887
3	U1	*Acinetobacter ursingii*	69	LC014147
		Uncultured from skin		KF083053
	L1	Uncultured *Streptococcus* from skin/nasopharynx	248	KF505347
	L2	*Enterobacter hormaechei*	4	KF516241
4	U1	*Granulicatella spp.*	>500	KJ575555
	U2	Uncultured *Pseudomonas spp.*	7	AY191342
		*Pseudomonas putida*	0	KP114213
	L1	*Streptococcus gordonii* (from infective endocarditis)	>500	KJ170416
	L2	*Streptococcus mitis*	>500	KP233800
		*Streptococcus oralis*		LN589729
	L4	Uncultured human mouth	333	JQ457994
		*Streptococcus sanguinis*		AY944229

^1^ Representative entries from the match listing

^2^ Number of BLAST sequences with 100% match (160 bp)

^3^ GenBank Accesion numbers

^4^ 0 indicates 99% match (159/160)

## DISCUSSION

In this study we have prepared dentine samples in a manner similar to that done for *in vitro* invasion investigations^16^ ^,^ ^22^ of dentine infection by pure cultures of bacteria such as *E. faecalis* and *Streptococcus* species. These approaches have been undertaken to study the mechanisms involved in dentinal tubule infection, and to investigate the effects of various antiseptics, irrigants, and antimicrobials in preventing dentinal tubule infection. Perhaps one limitation of such *in vitro* analyses is that they have been undertaken under conditions that are quite different from those that would be encountered *in vivo*. These include, for example, the presence of whole saliva, salivary flow, shear and abrasion, and nutrient pulses. Our studies here show that it is possible to readily achieve dentine infection *in vivo* to the levels and extent that can be obtained *in vitro* ^16^. This model therefore would be useful for testing the effects of new dentinal tubule occluding compounds^27^ or agents for preventing root caries^30^ in order to complement the *in vitro* experiments that have been previously employed.

Under laboratory conditions, Gram-positive cocci readily penetrate dentinal tubules. Historically, *E. faecalis* has been considered as a major invader of dentine^13^ ^,^ ^15^, but more recent molecular studies that do not employ cultivation methods suggest that *E. faecalis* may not be so prevalent as generally believed^23^. Invasions of dentine have been shown to contain a complex microbiota of Gram-positive and mainly Gram-negative bacteria^8^. Penetration of dentine by Gram-negative bacteria *in vitro* has not been investigated in such detail. Interestingly, periodontal bacteria *Porphyromonas gingivalis* were found to be unable to invade dentine unless co-cultured with *Streptococcus* ^16^. In this present article we have demonstrated microscopically, and by molecular means, that dentine *in vivo* can be invaded by Gram-negative bacteria, principally Gram-negative rods. Some of the organisms identified, e.g., *Enterobacter*, *Klebsiella,* seemed on first impression to perhaps be unusual. However, *Enterobacter* and *Klebsiella* species have been identified within the subgingival microbiota^8^. More recently, *Klebsiella* was identified in deep carious lesions underneath restorations^20^ and *E. hormaechei* was cultivated from human atherosclerotic tissue^24^. Members of the Enterobacteriaceae and Pseudomonadaceae are also found on the human tongue^9^. Our work thus provides further evidence that these Gram-negative organisms are found in the oral cavity and have the ability to penetrate dentine.

A range of bacterial species were present within a small number of dentine samples analyzed. Three samples showed disintegration of tubule structure, most likely arising from the lengthy preparation process (fixation, demineralization, dehydration, sectioning). Only a limited number of specimens were employed here because we were interested in first establishing a model system. The results suggest that the model can be applied to future studies of dentine hypersensitivity agents, determining their clinical efficacy and their ability to occlude tubules and block bacterial invasion^27^. It is acknowledged that the molecular methods used here do not differentiate between live or dead bacteria. However, it might be possible to utilize dentine discs, fracture them, and stain the intra-tubular bacteria with LIVE/DEAD stain. This method has recently been described in studies evaluating *in vitro* the antimicrobial effect of a commercial product on residual bacteria in dentinal tubules^11^.

One of the samples in the study described here was invaded by several species of Gram-positive cocci, which corroborates the notion that these organisms are often some of the first to invade dentine^15^. However, *E. faecalis* was not found in our analyses. We identified *Granulicatella*, *S. oralis*, *S. mitis,* and *S. gordonii* which, with the exception of *Granulicatella*, have been previously implicated in tubule invasion^15^. In addition, all of these bacteria including *Granulicatella* are organisms that have been linked with infective endocarditis. Therefore, there could potentially be an association between ability to invade dentine and ability to cause endocardial or intravascular infections^7^.

We have thus identified organisms that were present within dentinal tubules that have been exposed to many hundreds of different bacteria *in vivo* ^19^. In this study we only utilized four dentine samples to identify bacteria types that could invade the specimens under the condition used. Future clinical studies for testing efficacy of compounds or products in occluding tubules and preventing bacteria invasion would definitely employ many more subjects to provide suitable power. However, the molecular studies cannot be directly related to the morphological studies at this stage. We have established though that it is feasible to extract bacterial DNA from decalcified dentine. Our methodology would tend to identify the most prevalent microorganisms that were present within the dentine samples analyzed. We would like to develop these studies further in such a way that we could visualize and identify, by molecular techniques, the bacteria that have invaded the same dentine sample. This could be achieved by extracting bacterial DNA, or by detecting DNA using fluorescent in situ hybridization (FISH), from adjacent sections to those histochemically stained.

## CONCLUSION

In summary, this study has established a novel *in vivo* model for studying the infection of dentine by oral microorganisms. Dentine specimens exposed to the human oral environment become infected with microorganisms to similar extent and depth to dentine infected *in vitro* under laboratory conditions. In addition to streptococci, bacteria from the genera *Enterobacter*, *Klebsiella* and *Pseudomonas* were identified as primary invading organisms. This *in vivo* model should provide the means to confirm *in vitro* experimental results on the effects of antiseptics, irrigants, or tubule occluding agents on dentine invasion by oral bacteria.
